# Valosin Containing Protein as a Specific Biomarker for Predicting the Development of Acute Coronary Syndrome and Its Complication

**DOI:** 10.3389/fcvm.2022.803532

**Published:** 2022-03-18

**Authors:** Chenchao Xu, Bokang Yu, Xin Zhao, Xinyi Lin, Xinru Tang, Zheng Liu, Pan Gao, Junbo Ge, Shouyu Wang, Liliang Li

**Affiliations:** ^1^Department of Forensic Medicine, School of Basic Medical Sciences, Fudan University, Shanghai, China; ^2^Department of Cardiology, Shanghai Institute of Cardiovascular Diseases, Zhongshan Hospital, Fudan University, Shanghai, China

**Keywords:** valosin-containing protein, acute coronary syndrome, ventricular dysfunction, prognosis prediction, serological biomarker

## Abstract

**Background:**

Acute coronary syndrome (ACS) consists of a range of acute myocardial ischemia-related manifestations. The adverse events of ACS are usually associated with ventricular dysfunction (VD), which could finally develop to heart failure. Currently, there is no satisfactory indicator that could specifically predict the development of ACS and its prognosis. Valosin-containing protein (VCP) has recently been proposed to protect against cardiac diseases. Hence, we aimed to assess whether VCP in serum can serve as a valuable biomarker for predicting ACS and its complication.

**Methods:**

Human serum samples from 291 participants were collected and classified into four groups based on their clinical diagnosis, namely healthy control (*n* = 64), ACS (*n* = 40), chronic coronary syndrome (CCS, *n* = 99), and nonischemic heart disease (non-IHD, *n* = 88). Clinical characteristics of these participants were recorded and their serum VCP levels were detected by enzyme-linked immunosorbent assay (ELISA). Association of serum VCP with the development of ACS and its complication VD was statistically studied. Subsequently, GWAS and eQTL analyses were performed to explore the association between *VCP* polymorphism and monocyte count. A stability test was also performed to investigate whether VCP is a stable biomarker.

**Results:**

Serum VCP levels were significantly higher in the ACS group compared with the rest groups. Besides, the VCP levels of patients with ACS with VD were significantly lower compared to those without VD. Multivariate logistic regression analysis revealed that VCP was associated with both the risk of ACS (*P* = 0.042, OR = 1.222) and the risk of developing VD in patients with ACS (*P* = 0.035, OR = 0.513) independently. The GWAS analysis also identified an association between *VCP* polymorphism (rs684562) and monocyte count, whereas the influence of rs684562 on *VCP* mRNA expression level was further verified by eQTL analysis. Moreover, a high stability of serum VCP content was observed under different preservation circumstances.

**Conclusion:**

Valosin-containing protein could act as a stable biomarker in predicting the development of ACS and its complication VD.

## Introduction

Acute coronary syndrome (ACS) represents a series of acute myocardial ischemia-related symptoms caused by disruption of coronary artery plaque and consequent thrombosis-induced severe coronary artery stenosis or occlusion (1, 2). Depending on its severity, ACS could mainly lead to three manifestations: unstable angina pectoris, acute myocardial infarction (AMI), and sudden cardiac death (SCD) (3, 4). Given its high morbidity and mortality, ACS has long been considered as a life threat and a great burden to healthcare system worldwide. It is estimated that 40% of patients who experienced such a coronary event may die within 5 years. Moreover, the risk of death for those who suffered from recurrent cardiac events could be 5–6 times higher than general population (5–7).

Previous studies have demonstrated that the adverse prognostic events of ACS are mainly associated with ventricular dysfunction (VD), which may finally lead to heart failure of the patients (8, 9). Hence, the early treatment of ACS, which includes thrombolytic therapy and anticoagulant therapy, could be essential for better prognosis (10). To achieve that purpose, accurate indicators for the early diagnosis and prognosis estimation are required. Cardiac biomarkers, such as creatine kinase MB (CK-MB) and cardiac troponin T (cTnT), have been proved to be useful in predicting ACS, particularly non-ST segment elevated myocardial infarction (non-STEMI) (11–13). Due to the application of these biomarkers, the diagnostic efficiency of ACS has been significantly improved (14, 15). However, most of these biomarkers are not specific indicators for ACS, or rather a reflection of the established fact of myocardial infarction (16–21). Moreover, estimation of the long-term outcomes, which can be negatively affected by VD, remains to be further improved due to the lack of valid prognostic indicators. Therefore, an effective indicator that can be used to predict both the development of ACS and its complication in a convenient way is urgently needed.

Valosin-containing protein (VCP), also known as p97, is a conserved type II AAA+ (ATPases associated with diverse cellular activities) family protein abundantly expressed in cardiac tissues. The biological functions of VCP range from protein metabolism to intracellular homeostasis. The disruption of protein homeostasis has been proved to involve in various cardiac diseases, which includes heart failure, myocardial infarction, and diabetic cardiomyopathy (22). As an important maintainer of protein homeostasis in cardiovascular system, VCP has been suggested to have a protective role toward ischemia–reperfusion injury and VD (23–25). In our recent study, a significant increase in serum VCP concentration was observed in early myocardial ischemia-induced SCD cases, which further verified the activation of VCP expression toward acute myocardial ischemia (26). In addition, experiments on transgenic mice revealed that disruption of VCP activity could lead to the development of cardiomyopathy and defects in cardiomyocyte nuclear morphology, which suggests the pleiotropic functions of VCP in cardiac homeostasis (27). Though VCP has been considered to be a crucial cardiac protective factor, it remains obscure whether VCP is related to the development of VD in patients with ACS. Besides, the content changes of secretory VCP in ACS also deserve to be clarified.

In this research, the serum VCP levels among several different groups, namely healthy control group, ischemic heart disease (IHD) group, and non-IHD group, were investigated. For a better comparison, the IHD group was further subdivided into ACS group and chronic coronary syndrome (CCS) group. In addition, association between serum VCP levels and the development of ACS, along with its most common complication—VD—were statistically analyzed. The aim was to explore whether VCP could serve as a specific biomarker to predict both ACS and its complication.

## Materials and Methods

### Ethical Statement

Human serum samples were collected from participants who were admitted to Zhongshan Hospital affiliated to Fudan University (Shanghai, China) during the period August 2020 to August 2021. Sampling and study design was in agreement with the ethical principles stated in the Declaration of Helsinki of the World Medical Association (28) and approved by the Ethics Committee at Zhongshan Hospital, Fudan University (approval number: B2020-078R).

### Sample Classification

Human serum samples from 291 participants were collected and classified into four groups: healthy control group (*n* = 64), ACS group (*n* = 40), CCS group (*n* = 99), and non-IHD group (*n* = 88). Grouping was performed based on symptoms, diagnosis, medical history, and laboratory information. Individuals with none or mild diseases irrelevant to cardiac function were allocated into healthy control group. Individuals with IHD were subdivided into ACS and CCS groups according to the ESC and JCS guidelines (3, 29). The rest cases, which include arrhythmia, valvular heart disease, congenital heart disease, and cardiomyopathy, were pooled into non-IHD group. Demographic information of the donors, which includes age, sex, history of smoking, and drinking, and also their echocardiographic parameters and blood–urine biochemistry test results were collected. For the purpose of this study, whether patients developed VD following diagnosis of ACS was also retrieved from medical records. Based on the clinical practice, the diagnosis of VD is based on interpretation of combined qualitative and quantitative echocardiographic parameters by an experienced operator who classifies the cardiac function as “normal” and “dysfunction” (30). Patients who developed VD preceding the diagnosis of ACS were excluded from this study. Depending on the presence of VD, the ACS group was subdivided into two cohorts, namely “ACS + VD” (*n* = 14) and “ACS + non-VD” (*n* = 26). Serum samples were collected immediately after admission before the first medical intervention.

### Preparation of Serum Samples

A total of 5 mL peripheral blood was acquired from each donor and coagulated at room temperature (RT) for 30 min, followed by centrifugation at 12,000 rpm for 10 min at 4°C. Separated serum samples were stored at −80°C until use.

### Serum VCP Level Detection

Samples were initially diluted with saline by a serial dilution of 2-, 5-, 10-, and 100-folds. The preliminary experiment suggests that a dilution of 5-folds yielded good results within the range of standards. Hence, all serum samples were diluted in a ratio of 1:5 for further assay in this study. Procedure of the two-site sandwich assay is demonstrated as previously described (26). VCP was separated by binding to the corresponding dendrimer-linked monoclonal antibody on the microplate. Additionally, a horse radish peroxidase (HRP)-conjugated secondary monoclonal antibody was applied to form an antibody-antigen-labeled antibody sandwich. Finally, unbound, labeled antibody was removed by elution.

The serum VCP detection was conducted using a commercial kit from Shanghai YuBo Biotechnology (catalog no.: YB-VCP-Hu, Shanghai, China) for enzyme-linked immune sorbent assay (ELISA) purposes. Briefly, an aliquot of 50 μL diluted sample or the standards was added to a 96-well microplate and incubated with 100 μL rabbit antimouse HRP-conjugated secondary antibody at 37°C for 1 h. Subsequently, the unbound antibody was washed for five times. The microplates were then incubated with 100 μL/well enzyme substrates and kept in dark at 37°C to allow immunoreaction. Finally, the reaction was quenched by the addition of 50 μL stop solution. Signal intensity was detected of the absorbance at the wavelength of 450 nm using the BioTek Epoch Microplate Spectrophotometer (Biotek, Winooski, VT, USA). The concentration of VCP in each sample was determined based on the calibration curve generated with the human full-length VCP protein standard. Sample diluent was made of 10% NBS, 0.05% Tween-20, 0.2% procline-300, and PBS with pH 7.2–7.4. As per the manufacturer's instructions, crossreaction with other nonspecific analytes and the influence of a spectrum of other biological substances and drugs were negligible due to the use of specific monoclonal antibodies in this system. The specificity of this commercial kit was also verified in our previous study (26).

### Stability Test

To test the stability of VCP in human serum, two groups of serum samples were exposed at 4°C or RT for up to 6 days, respectively. Meantime, aliquots from 6 time points (1, 3, 6 h, 1, 3, and 6 d) were collected for VCP assay. Serum samples in both two groups were collected from two individuals and treated under 3 different conditions: (A) original serum sample without treatment, (B) original serum sample supplemented with 1,500 pg/mL human VCP standard, (C) original serum sample supplemented with 750 pg/mL human VCP standard. In addition, a third group that consists of serum samples from another four individuals was applied to investigate the possible effect of multigelation. Specifically, five aliquots from each sample were thawed and frozen repeatedly for 1 to 5 times, respectively. In each thaw–frozen cycle, samples were frozen at −20 °C for 20 min and then brought back to RT for 1 h.

### Genome-Wide Association Study (GWAS) Analysis of *VCP* Polymorphisms and Monocyte Count

Genome-wide association study summary data of 145 K individuals from the UK Biobank release (https://www.ukbiobank.ac.uk/) were used to explore the association between the *VCP* polymorphisms and monocyte count. Specifically, variants with a minor allele frequency greater or equal to 1% and an imputation info score greater or equal to 0.5 were kept for association analysis. A Bonferroni-corrected *p*-value of 5.00E-8 was used as the threshold to assess the statistical significance. Eventually, variants that reached genome-wide significance were annotated using HaploReg v4.1 (31). The regional plot was generated using LocusZoom (https://my.locuszoom.org/).

### Expression Quantitative Trait Loci (EQTL) Analysis of Rs684562 and *VCP* MRNA Expression Level

To further explore the association between *VCP* mRNA expression levels and the polymorphism identified by GWAS, an eQTL analysis was performed with R software (version 4.0.5) by employing the 1000 Genomes Project Phase 3 genotype data from the Ensembl database (http://www.ensembl.org/) and the deep RNA-sequencing data of lymphoblastoid cell lines (LCLs) collected from 462 individuals of five populations genotyped in the 1000 Genomes Project (32, 33).

### Statistical Analysis

Statistical analyses were performed with R software (version 4.0.5) and GraphPad Prism software v 8.3.0 (La Giolla, CA, USA). Continuous variables, such as serum VCP levels, were presented as mean ± standard error of mean (SEM). Categorical variables, such as female gender, were exhibited as numbers or proportions. Differences among groups were compared using one-way ANOVA test for normally distributed continuous variables with homogeneous variance; otherwise, Kruskal–Wallis test were used. Pearson's chi-squared test was used for categorical variables. For comparisons between two groups, parametric Student's *t*-test or nonparametric Mann–Whitney *U*-test was performed, which depends on the group size. Univariate logistic regression analysis was initially performed to screen risk factors for ACS or VD in patients with ACS. Variables with significance were further investigated with multivariate logistic stepwise regression analysis. All statistical tests were two-sided, and *p* < 0.05 was considered statistically significant.

## Results

### Clinical Characteristics of Study Population

Of the 291 participants enrolled in this study, no significant difference was observed among different groups concerning their gender, drinking habit, and the blood level of some biochemistry indicators, such as hemoglobin, creatine, eGFR, HbA1c, triglyceride, and urine pH. However, there was statistically significant difference in terms of their age, smoking habit, along with the level of the rest biochemistry indicators. The blood levels of several indicators, such as fasting plasma glucose (FPG), cTnT, N-terminal pro-B-type natriuretic peptide (NT-proBNP), CK-MB, creatine kinase MM (CK-MM), and high-sensitive C-reactive protein (hs-CRP), were substantially raised in ACS group compared to the rest groups. Meanwhile, the glycosylated albumin (GA-L) level was remarkably raised in non-IHD, CCS, and ACS groups, whereas the total cholesterol (TC), low-density lipoprotein cholesterol (LDL-C), and high-density lipoprotein cholesterol (HDL-C) levels were reduced in these three groups (Table 1). In terms of echocardiographic parameters, patients with ACS exhibited significantly reduced left ventricular ejection fraction (LVEF) compared to the rest groups. Besides, increased left atrium diameter (LAD) and left ventricular end diastolic diameter (LVDd) were observed in ACS, CCS, and non-IHD groups, compared to the healthy control group (Table 2).

**Table 1:** Demographic and laboratory information of the study population.

	**Control (*n* = 64)**	**Non-IHD (*n* = 88)**	**CCS (*n* = 99)**	**ACS (*n* = 40)**	***P*** **-value**
**Demographic characteristic**						
Age (years)	58 (48.0–68.0)	63 (51.8–71.3)	67 (60.3–72.0)	67 (57.5–75.3)	**0.002**
Gender (female%)	23 (35.9)	32 (36.4)	32 (32.7)	7 (17.5)	0.168
Smoking [*n*(%)]	2 (3.1)	19 (21.6)	22 (22.4)	15 (37.5)	**<0.001**
Drinking [*n*(%)]	1 (1.6)	9 (10.2)	11 (11.2)	4 (10.0)	0.095
**Biochemistry indicators**						
Hemoglobin (g/L)	130 (118.0–149.0)	124 (106.8–135.0)	127 (104.3–138.8)	125 (108.0–134.0)	0.095
Albumin (g/L)	45 (38.0–48.0)	40 (36.0–42.0)	39 (37.0–42.8)	36 (34.0–41.0)	**<0.001**
Creatinine	80 (66.8–102.0)	84 (73.8–115.0)	87 (68.3–113.8)	89 (76.0–124.8)	0.522
eGFR (mL/min/1.73 m^2^)	90 (44.0–102.0)	77 (53.5–96.5)	74 (52.3–94.8)	77 (48.8–88.3)	0.404
FPG (mmol/L)	5.2 (4.8–5.9)	5.4 (4.7–7.0)	5.6 (4.9–7.4)	6.7 (5.4–9.1)	**0.002**
GA-L (%)	13.3 (12.0–15.5)	15.5 (13.0–18.6)	15.1 (13.4–16.6)	15.1 (13.7–19.3)	**0.001**
HbA1c (%)	5.7 (5.4–5.9)	5.9 (5.4–7.1)	5.8 (5.6–7.1)	6.0 (5.6–7.8)	0.094
cTnT (ng/mL)	0.012 (0.006–0.049)	0.043 (0.014–0.124)	0.040 (0.010–0.121)	0.536 (0.075–1.842)	**<0.001**
Log(NT-proBNP)(pg/mL)	4.947 (3.694–6.280)	6.963 (6.076–7.935)	6.572 (4.908–7.886)	7.477 (6.482–8.765)	**<0.001**
CK-MB (U/L)	15 (12.0–20.0)	15 (12.0–18.0)	15 (13.0–22.0)	20 (14.8–44.3)	**0.001**
CK-MM (U/L)	62 (41.0–96.0)	59 (34.0–108.0)	52 (33.3–83.8)	171 (44.5–488.5)	**0.004**
hs-CRP (mg/L)	1.1 (0.4–3.2)	2.0 (0.6–10.6)	1.4 (0.5–8.3)	6.4 (1.9–36.3)	**<0.001**
TC (mmol/L)	4.930 (4.165–5.490)	3.780 (3.220–4.610)	3.310 (2.870–3.890)	3.410 (2.850–4.170)	**<0.001**
Triglyceride (mmol/L)	1.480 (1.095–2.078)	1.260 (0.907–1.755)	1.310 (0.930–1.940)	1.190 (0.780–1.910)	0.320
LDL-C (mmol/L)	2.795 (1.990–3.327)	2.070 (1.640–2.810)	1.620 (1.290–2.130)	1.560 (1.340–2.220)	**<0.001**
HDL-C (mmol/L)	1.210 (1.012–1.490)	1.010 (0.860–1.250)	1.040 (0.840–1.160)	1.030 (0.830–1.170)	**0.004**
UA	328(265–409)	379(302–483)	339(291–399)	398(314–462)	**0.003**
Urine pH	5.5 (5.5–6.1)	6.0 (5.5–6.0)	5.5 (5.5–6.5)	5.5 (5.1–6.0)	0.637
Proteinuria					**0.001**
–	38 (66.7)	69 (83.1)	73 (80.2)	19 (50.0)	
+	10 (17.5)	8 (9.6)	10 (11.0)	14 (36.8)	
≥++	9 (15.9)	6 (7.2)	8 (8.8)	5 (13.2)	

*eGFR, estimated glomerular filtration rate; FPG, fasting plasma glucose; GA-L, glycosylated albumin; HbA_1c_, glycosylated hemoglobin; cTnT, cardiac troponin T; NT-proBNP, amino terminal probrain natriuretic peptide; CK-MB, creatine kinase MB; CK-MM, creatine kinase MM; hs-CRP, high-sensitivity C-reactive protein; TC, total cholesterol; LDL-C, low-density lipoprotein cholesterol; HDL-C, high-density lipoprotein cholesterol; UA, uric acid. Bold value indicates the statistical significance*.

**Table 2:** Medical history and echocardiography of the study population.

	**Control (*n* = 64)**	**Non-IHD (*n* = 88)**	**CCS (*n* = 99)**	**ACS (*n* = 40)**	***P*** **-value**
**History of cardiovascular disease**						
Arrhythmia	0 (0.0)	52 (59.1)	23 (23.2)	5 (12.5)	**<0.001**
Cardiomyopathy	0 (0.0)	9 (10.2)	7 (7.1)	0 (0.0)	**0.009**
Valvular disease	0 (0.0)	29 (33.0)	5 (5.1)	2 (5.0)	**<0.001**
Congenital heart disease	0 (0.0)	11 (12.5)	0 (0.0)	0 (0.0)	**<0.001**
Hypertension	28 (43.8)	38 (43.2)	71 (72.4)	30 (75.0)	**<0.001**
Dyslipidemia	28 (43.8)	38 (43.2)	71 (72.4)	30 (75.0)	**<0.001**
Diabetes	7 (10.9)	3 (3.4)	7 (7.1)	4 (10.0)	0.298
**Medication received before admission**						
ACEI/ARBs	6 (9.4)	19 (22.1)	39 (40.6)	16 (40.0)	**<0.001**
CCB	11 (17.2)	22 (25.0)	29 (30.2)	11 (27.5)	0.314
β blockers	6 (9.4)	23 (26.1)	37 (38.5)	14 (35.0)	**0.001**
diuretics	4 (6.2)	29 (33.0)	29 (30.2)	5 (12.5)	**<0.001**
Digoxin	0 (0.0)	7 (8.0)	5 (5.2)	0 (0.0)	**0.040**
Statins	12 (18.8)	5 (5.7)	52 (54.2)	21 (52.5)	**<0.001**
Antithrombotics	7 (10.9)	12 (13.6)	72 (75.0)	34 (85.0)	**<0.001**
Anticoagulation	2 (3.1)	22 (25.0)	10 (10.4)	9 (22.5)	**0.001**
Nitrate esters	1 (1.6)	9 (10.2)	18 (18.8)	18 (45.0)	**<0.001**
Trimetazidine	0 (0.0)	2 (2.3)	3 (3.1)	0 (0.0)	0.530
Insulin	2 (3.1)	6 (6.8)	14 (14.6)	1 (2.5)	**0.033**
OAD	2 (3.1)	12 (13.6)	12 (12.5)	6 (15.0)	0.090
**Echocardiographic parameters**						
ARD (mm)	34 (31–37)	33 (32–37)	33 (31–35)	34 (32–36)	0.324
LAD (mm)	38 (36–40)	44 (41–50)	41 (39–45)	41 (38–42)	**<0.001**
LVDd (mm)	46 (44–49)	49 (45–54)	48 (44–52)	49 (45–55)	**0.005**
IVS (mm)	10 (9–11)	10 (9–12)	10 (9–12)	10 (10–12)	0.068
LVPW (mm)	9 (9–10)	10 (9–11)	10 (9–10)	10 (9–10)	0.345
PAP (mmHg)	30 (30–34)	35 (31–44)	33 (31–38)	32 (30–35)	**<0.001**
LVEF (%)	65 (63–67)	64 (58–66)	60 (50–66)	54 (43–62)	**<0.001**

*ACEI, angiotensin-converting enzyme inhibitors; ARB, angiotensin receptor blockers; CCB, calcium channel blocker; OAD, oral antidiabetic drugs; ARD, aortic root diameter; LAD, left atrial diameter; LVDd, left ventricular end diastolic diameter; IVS, interventricular septal thickness; LVPW, posterior wall thickness of left ventricle; PAP, pulmonary arterial systolic pressure; LVEF, left ventricular ejection fraction. Bold value indicates the statistical significance*.

### Significantly Increased Serum VCP Level in ACS Group

**Figure 1:**
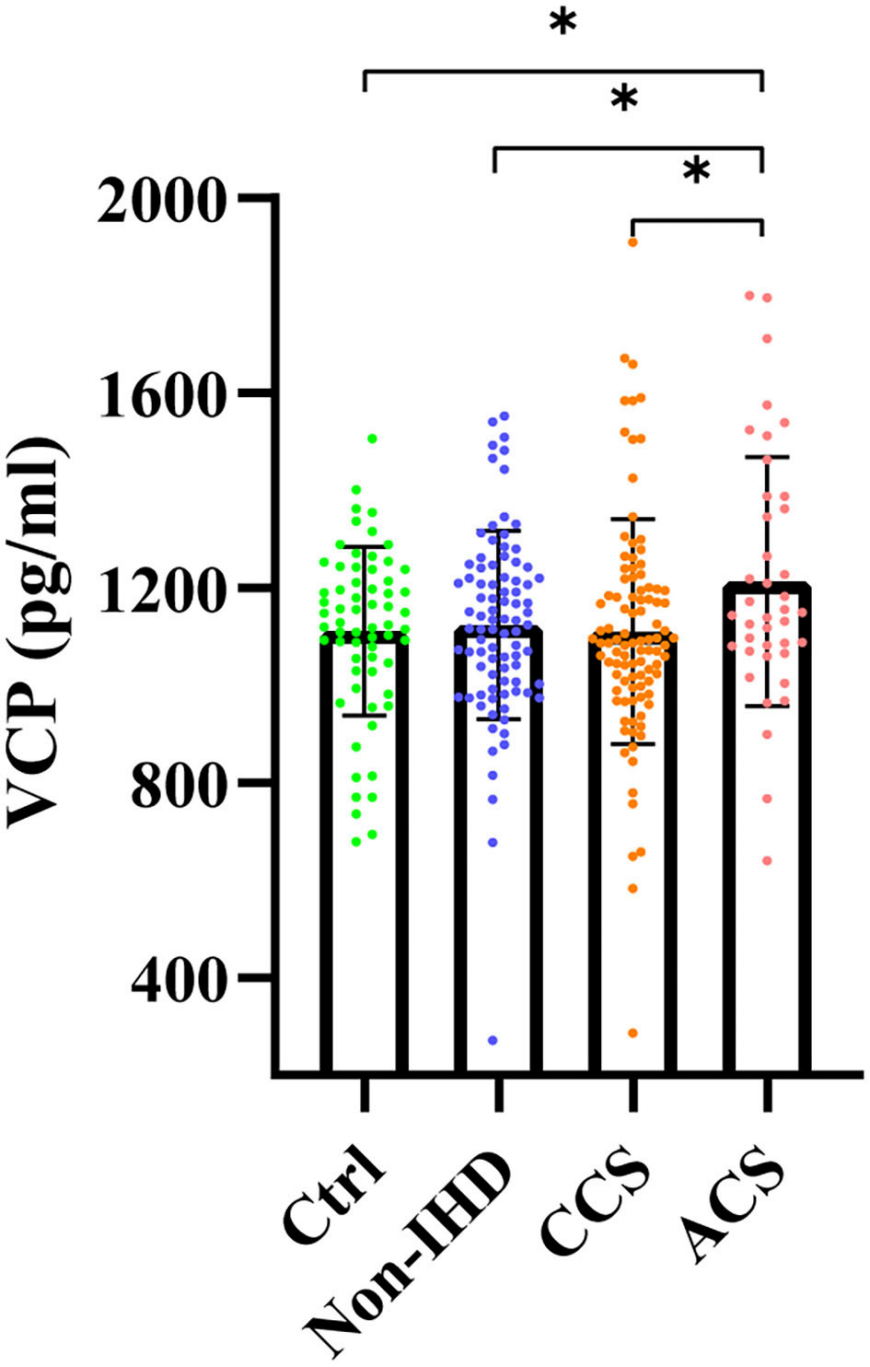
Serum VCP levels of healthy control group (Ctrl), nonischemic heart disease group (non-IHD), chronic coronary syndrome group (CCS), and acute coronary syndrome group (ACS) (*p < 0.05).

Serum VCP levels of the four groups were exhibited in Figure 1. Mean ± SEM of the VCP level for each group was also plotted (ACS: 1,213.00 ± 40.36, Ctrl: 1,111.00 ± 21.62, non-IHD: 1,124.00 ± 20.66, CCS: 1,111.00 ± 23.23). As can be seen, serum VCP level is significantly increased in the ACS group compared with the healthy control group (*P* = 0.030), the non-IHD group (*P* = 0.031), and the CCS group (*P* = 0.023), whereas there was no significant difference neither between the healthy control group and the non-IHD group (*P* = 0.680), or between the healthy control group and the CCS group (*P* = 0.987). Besides, there is also no significant difference between the CCS group and the non-IHD group (*P* = 0.677).

### Statistical and Genetic Association Between VCP and ACS

According to the univariate logistic regression analysis based on data from all 291 participants, the odds ratios (OR) with its 95% confidence intervals (CIs) for 36 possible indicators are presented in Table 3. As can be seen, VCP, along with several other indicators, which include FPG, cTnT, NT-proBNP, CK-MB, CK-MM, uric acid (UA), and proteinuria (+), is positively correlated with the risk of ACS, whereas albumin and LVEF were negatively correlated with the risk of ACS. To further verify the association between VCP and the risk of ACS, a multivariate logistic stepwise regression analysis was performed for the above 10 identified indicators. The statistical results revealed that VCP, along with cTnT and UA, was independent variables in the optimized model (Table 4). Taken together, these findings suggest that VCP level is strongly related to the risk of ACS.

**Table 3:** Univariate logistic regression analysis of ACS and possible indicators (all indicators increase by 1 unit unless otherwise specified).

**Indicator**	**OR (95% CI)**	***P-*** **value**
VCP^a^	1.234 (1.044, 1.459)	**0.014**
Age	0.999 (0.968, 1.032)	0.975
Gender	0.437 (0.175, 1.096)	0.078
Smoking	2.073 (0.934, 4.598)	0.073
Drinking	0.879 (0.262, 2.943)	0.834
Hemoglobin	0.996 (0.981, 1.012)	0.647
Albumin	0.923 (0.854, 0.998)	**0.045**
Creatinine	1.001 (0.999, 1.003)	0.487
eGFR	0.996 (0.983, 1.009)	0.508
FPG	1.113 (1.003, 1.235)	**0.045**
GA-L	1.038 (0.958, 1.124)	0.361
HbA1c	1.045 (0.775, 1.410)	0.772
cTnT^b^	1.052 (1.018, 1.088)	**0.003**
log(NT-proBNP)	1.353 (1.103, 1.659)	**0.004**
CK-MB	1.034 (1.011, 1.058)	**0.004**
CK-MM	1.003 (1.001, 1.004)	**0.003**
hs-CRP	1.009 (1.000, 1.019)	0.062
TC	1.177 (0.752, 1.844)	0.476
Triglyceride	1.089 (0.776, 1.529)	0.622
LDL-C	1.155 (0.685, 1.948)	0.590
HDL-C	0.592 (0.204, 1.717)	0.334
UA	1.004 (1.001, 1.006)	**0.007**
Urine pH	0.709 (0.417, 1.205)	0.204
Proteinuria +^c^	5.379 (2.068, 13.988)	**<0.001**
Proteinuria ≥++^d^	2.401 (0.705, 8.183)	0.161
Hypertension	1.141 (0.492, 2.647)	0.759
Dyslipidemia	1.444 (0.399, 5.235)	0.576
Diabetes	1.292 (0.614, 2.718)	0.500
ARD	1.093 (0.986, 1.212)	0.090
LAD	0.955 (0.887, 1.028)	0.219
LVDd	1.037 (0.989,1.087)	0.137
LVDs	1.037 (0.993, 1.082)	0.099
IVS	1.037 (0.850, 1.266)	0.719
LVPW	1.000 (0.843, 1.185)	0.997
PAP	0.974 (0.923, 1.028)	0.335
LVEF	0.969 (0.940, 0.998)	**0.037**

a *VCP increases by 100 units*.

b *cTnT increases by 0.003 units*.

c *Compared with nonproteinuria group*.

d *Compared with nonproteinuria group*.

*Bold value indicates the statistical significance*.

**Table 4:** Multivariate logistic stepwise regression analysis of ACS and candidate indicators (all indicators increase by 1 unit unless otherwise specified).

**Indicator**	**OR (95% CI)**	***P*** **value**
VCP^a^	1.222 (1.008, 1.482)	**0.042**
cTnT^b^	1.054 (1.015, 1.093)	**0.006**
UA	1.004 (1.001, 1.007)	**0.007**
FPG	1.121 (0.960, 1.310)	0.150
CK-MM	1.002 (0.998, 1.006)	0.286
CK-MB	1.014 (0.959, 1.073)	0.520
Albumin	0.957 (0.830, 1.113)	0.570
LVEF	1.014 (0.959, 1.073)	0.620
Proteinuria	1.193 (0.508, 2.804)	0.686
NT-proBNP	0.970 (0.616, 1.528)	0.896

a *VCP increases by 100 units*.

b *cTnT increases by 0.003 units*.

*Bold value indicates the statistical significance*.

**Figure 2:**
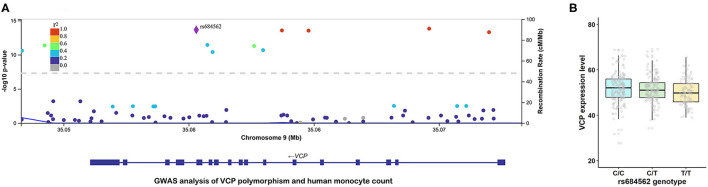
*VCP* polymorphism is correlated with human monocyte count and *VCP* mRNA expression level. **(A)** Association between rs684562 and human monocyte count revealed by GWAS (*P* = 2.20E-14). **(B)** Association between *VCP* mRNA expression level and the genotype of rs684562 revealed by eQTL analysis.

To further investigate the potential role of VCP in the occurrence of ACS at a genetic level, a GWAS analysis that focusses on genetic variants associated with monocyte count was performed. As shown in Figure 2A, a common SNP (rs684562) located on the *VCP* gene, which is indicated by a purple rhombic dot, was significantly associated with human monocyte count (*P* = 2.20E-14). In addition, the eQTL analysis further revealed that *VCP* mRNA level was associated with the genotype of rs684562 (Figure 2B). Individuals carrying the T allele on the rs684562 locus tend to have decreased *VCP* levels as compared to the C allele. Taken together, these findings imply that gene polymorphism might affect the VCP expression that consequently modulates peripheral blood monocyte count and inflammation and regulates the development of ACS.

### Decreased Serum VCP Level in Patients With ACS With VD

**Figure 3:**
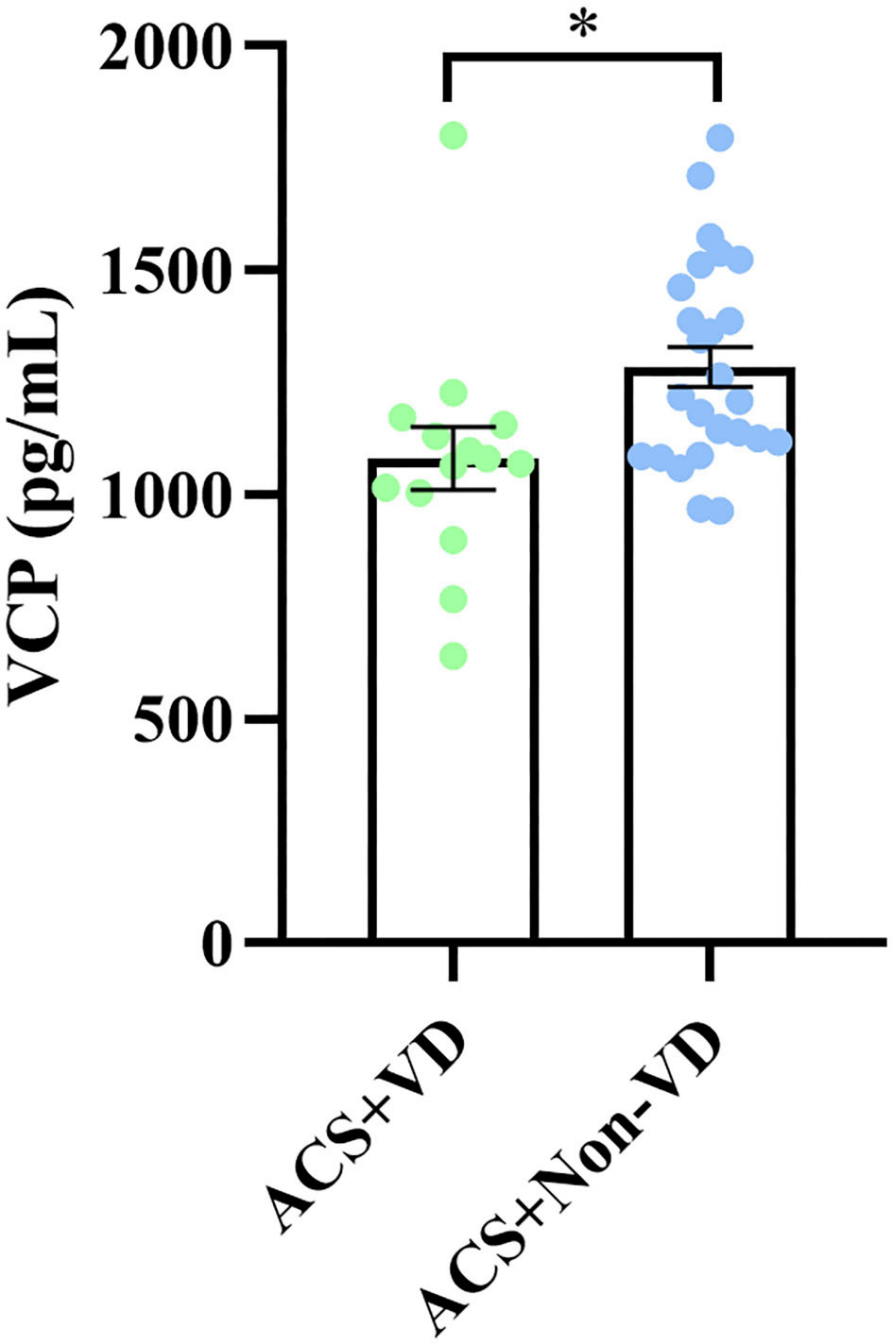
A significant difference in serum VCP level was observed between patients with ACS with VD and patients with ACS without VD (*p < 0.05).

Since the long-term prognosis of ACS is closely reflected by the presence of VD, we then subdivided the ACS group into 2 cohorts depending on whether or not the patient developed VD and then compared the serum VCP level between the two cohorts. Our results showed that patients with ACS who developed VD exhibited significantly decreased serum VCP levels compared to those who did not develop VD (*P* = 0.014) (Figure 3).

### Association Between VCP and the Risk of Developing VD in Patients With ACS

According to the univariate logistic regression analysis based on data from the 40 patients with ACS, the ORs with its 95% CI for 36 possible indicators are presented in Table 5. Among the indicators, only VCP, UA, left ventricular diameter in systole (LVDs), and interventricular septal thickness (IVS) are statistically correlated with the risk of developing VD in the ACS cohort.

**Table 5:** Risk factors for VD in patients with ACS evaluated by univariate logistic regression analysis (all indicators increases by 1 unit unless otherwise specified).

**Indicator**	**OR (95% CI)**	***P*** **-value**
VCP^a^	0.651 (0.442, 0.957)	**0.029**
Gender	0.256 (0.028, 2.383)	0.231
Age	1.033 (0.979, 1.089)	0.236
Smoking	0.889 (0.231, 3.425)	0.864
Drinking	0.590 (0.056, 6.266)	0.661
Hemoglobin	0.980 (0.951, 1.010)	0.199
Albumin	0.997 (0.866, 1.147)	0.966
Creatinine	1.000 (0.996, 1.003)	0.775
eGFR	0.983 (0.961, 1.006)	0.148
FPG	1.193 (0.993, 1.432)	0.059
GA-L	1.144 (0.968, 1.351)	0.114
HbA1c	1.833 (0.985, 3.414)	0.056
cTnT^b^	0.989 (0.975, 1.003)	0.129
log(NT-proBNP)	1.578 (0.987, 2.523)	0.057
CK-MB	0.973 (0.939, 1.009)	0.139
CK-MM	0.998 (0.996, 1.001)	0.176
hs-CRP	1.000 (0.985, 1.015)	0.976
TC	0.496 (0.201, 1.221)	0.127
Triglyceride	1.169 (0.720, 1.900)	0.528
LDL-C	0.270 (0.067, 1.080)	0.064
HDL-C	0.105 (0.005, 2.032)	0.136
UA	1.005 (1.000, 1.009)	**0.033**
Urine pH	0.784 (0.255, 2.412)	0.672
Proteinuria +	0.562 (0.194, 1.628)	0.288
Proteinuria ≥++	0.344 (0.032, 3.688)	0.378
Hypertension	1.351 (0.289, 6.320)	0.702
Dyslipidemia	0.590 (0.056, 6.266)	0.661
Diabetes	3.400 (0.873, 13.239)	0.078
ARD	1.224 (0.994, 1.508)	0.057
LAD	1.053 (0.913, 1.215)	0.480
LVDd	1.097 (0.999, 1.205)	0.052
LVDs	1.131 (1.028, 1.244)	**0.012**
IVS	0.507 (0.292, 0.879)	**0.016**
LVPW	0.641 (0.343, 1.200)	0.165
PAP	1.052 (0.952, 1.162)	0.318
LVEF	0.952 (0.900, 1.007)	0.086

a *VCP increases by 100 units*.

b *cTnT increases by 0.003 units*.

c *Compared with proteinuria negative group*.

d *Compared with proteinuria negative group*.

*Bold value indicates the statistical significance*.

To further confirm the association between VCP and the risk of developing VD, a multivariate logistic stepwise regression analysis was again performed for the above 4 identified indicators. Statistical results revealed that VCP, LVDs, and IVS were independent variables in the optimized model (Table 6), which suggest that VCP is significantly associated with the development of VD in patients with ACS. In addition, the OR (0.513) with its 95% CI (0.276–0.954) indicated that the risk of developing VD tends to reduce as the serum VCP level increases. Similarly, it can be inferred that increased IVS and decreased LVD are significantly related to reduced odds of developing VD in the patients with ACS.

**Table 6:** Risk factors for VD in patients with ACS evaluated by multivariate logistic stepwise regression analysis (all indicators increase by 1 unit unless otherwise specified).

**Indicator**	**OR (95% CI)**	***P*** **-value**
VCP^a^	0.513 (0.276, 0.954)	**0.035**
LVDs	1.315 (1.031, 1.677)	**0.028**
IVS	0.272 (0.094, 0.786)	**0.016**
UA	1.003 (0.996, 1.011)	0.394

a *VCP increases by 100 units*.

*Bold value indicates the statistical significance*.

### Stability of VCP in Serum

**Figure 4:**
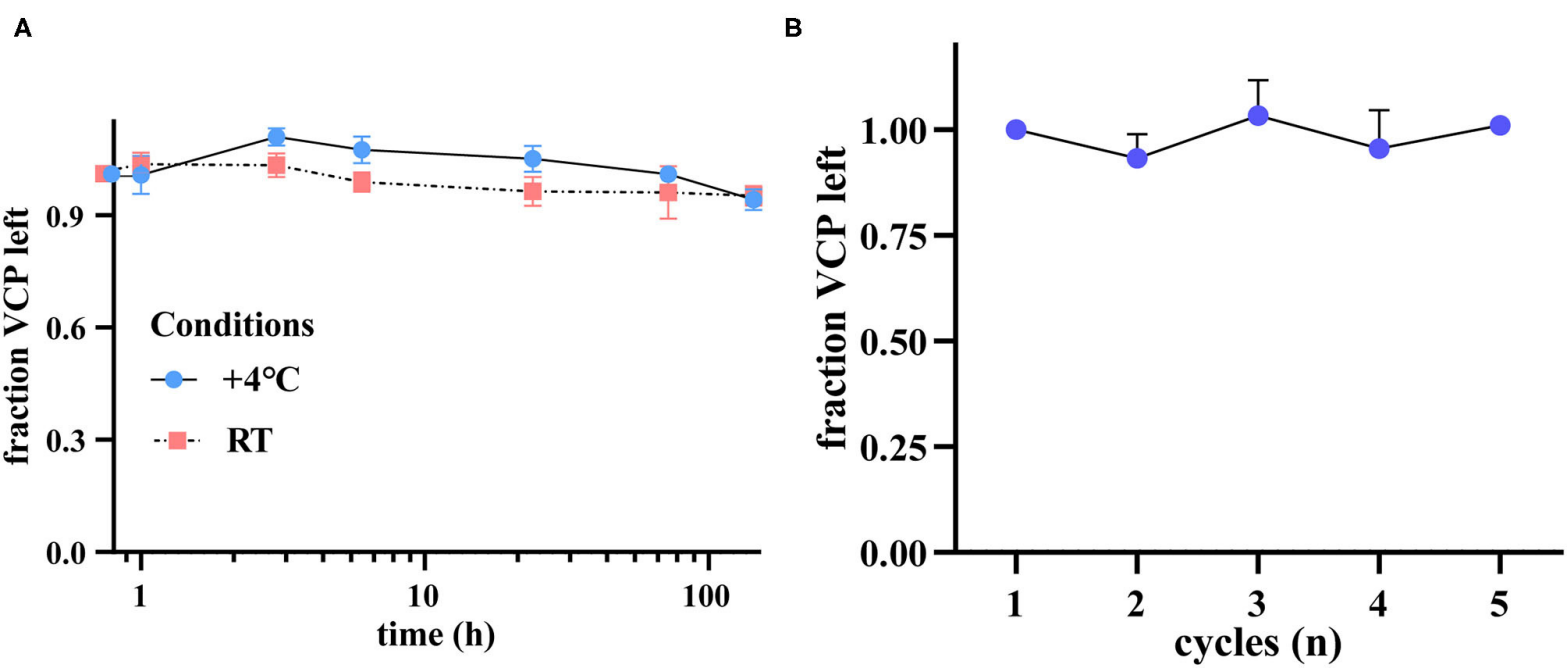
VCP is a stable serological biomarker under different preservation circumstances. **(A)** VCP content left after 1, 3, 6 h, 1, 3, and 6 d, illustrated by fraction change (*n* = 6). **(B)** VCP content left after 1–5 cycles of freezing and thawing (*n* = 4).

Given the fact that optimal storage and handling condition of serum samples cannot always be achieved during clinical practice, we have tested the stability of VCP upon three different circumstances. The results showed that after 6 days, the final VCP content slightly declined to 94.11% and 95.19% of its initial level when stored at 4°C or RT, respectively (Figure 4A). In addition, the VCP content in repeatedly thawed and frozen samples also maintained at a stable level after five freeze–thaw cycles (Figure 4B). These findings suggest that neither long-term storage at 4°C or RT nor multigelation could seriously affect serum VCP quantity.

## Discussion

In this study, serum VCP level was found to be significantly increased in the ACS group compared with the healthy control, CCS, and non-IHD groups. Meanwhile, statistical results from the multivariate logistic regression models also demonstrated that the serum VCP level is an independent risk factor for ACS (*P* = 0.042, OR = 1.222). More importantly, by dividing the ACS cohort into “ACS + VD” and “ACS + non-VD” subgroups, we have further observed that during progression of ACS, patients who developed VD exhibited significantly decreased serum VCP levels compared to those who did not develop VD. Multivariate logistic regression analysis showed that serum VCP was an independent variable that significantly correlated with the risk of VD in patients with ACS (*P* = 0.035, OR = 0.513). These outcomes suggest that VCP could be an effective biomarker for the prediction of both ACS and its complication.

To explore the possible mechanism behind the correlation between serum VCP level and the occurrence of ACS, a GWAS analysis that focusses on genetic variants associated with monocyte count was performed. Accordingly, rs684562, which is a common SNP located on the *VCP* gene, was found to be significantly associated with human monocyte count. The eQTL analysis further suggested that *VCP* mRNA expression level was associated with the genotype of rs684562, since individuals carrying the T allele tended to have a lower *VCP* level compared with those carrying the C allele. Inflammation with monocyte aggregation plays an important role in the development and progression of atherosclerosis and other cardiovascular diseases (34). It is now widely accepted that inflammation dysfunction is one of the main mechanisms of ACS (35). Thus, our findings indicated that the upregulation of VCP in ACS might be related to its participation in modulating monocyte count and inflammation. Moreover, previous studies have revealed that elevated levels of inflammatory markers produced by enhanced inflammation, along with increased white blood cell counts, are not only associated with a higher ACS incidence, but also a sign of poor prognostic events, which include VD and heart failure (36–39). According to a recent study, sufficient VCP in the heart could prevent pressure overload-induced heart failure by rectifying the inflammatory signaling and enhancing the cardiac resistance to oxidative stress (40). Therefore, the antiinflammatory function of VCP might explain its upregulation in the “ACS + non-VD” group. In addition, overexpression of VCP has been proposed to protect the cardiac function from negative effects of pathological Ca^2+^ overload by modulating Ca^2+^ uptake proteins (41), which we assume might also be a pathway for VCP to work against the development of VD.

Except for the predictive value of a biomarker, its stability is also a critical concern for clinical use. Our stability test results revealed that serum VCP level merely slightly decreased by 5.89 and 4.81% after 6 days when stored at 4°C or RT, respectively. This finding suggests that the stability of VCP is similar to several previously proposed biomarkers for the diagnosis of ACS, such as aspartate aminotransferase (AST) (reduced by 2.1%, 4°C, 7d), CK-MB (increased by 1.9%, 4°C, 7d) and cTnT (increased by 0.6%, 4°C, 7d), and better than some others, such as cardiac troponin I (cTnI) (reduced by 19.3%, 4°C, 7d) (42). Furthermore, no significant fraction reduction was observed in terms of the serum VCP level even after 5 freeze–thaw cycles. These indicate that VCP is stable in serum under different storage circumstances and thus could be applied as a suitable serological biomarker. During clinical practice, such kind of stable indicators should be of great value, as their expression levels in blood samples could be accurately quantified even after long-term storage.

Apart from VCP, some common indicators, which include albumin, FPG, UA, proteinuria, LVEF, cTnT, NT-proBNP, CK-MB, and CK-MM, were also identified to be the independent risk factors for ACS in this study. Among these indicators, the last four has previously been reported to be applied in diagnosis of ACS. From our laboratory test results, it can be observed that the serum levels of cTnT, CK-MB, and CK-MM were indeed notably elevated in the ACS group. However, previous studies also revealed their limitations in the diagnosis of ACS. Taking cTnT, the most representative one, for example, its elevation reflects the “fait accompli” of myocardial infarction, rather than progression of ACS (16, 17). As for the other indicators, their elevations are most likely to be physiological compensation effects, which are not specific secondary reactions to ACS. For example, an increased FPG level could be explained by hyperglycemia under the stress of ACS, known as stress induced hyperglycemia (SIH) (18, 19), whereas the elevation of UA is possibly due to the fact that UA level is closely related to oxidative stress, endothelial dysfunction, inflammation, and vasoconstriction, which are the common features in the episode of ACS (20, 21). In the recent years, the development of omics technology like metabolomics has also been proposed to promote the diagnosis of ACS. However, its clinical application has not yet been guaranteed due to problems concerning technical challenges and data interpretation (43). In view of feasibility, promising biomarkers that can be assayed using simple detection methods are currently still preferentially considered. Recently, serum CCL21 level was found to be independently associated with outcome after ACS (44), which has highlighted the potential of applying gene expression pattern to promote clinical evaluation. Similarly, the inclusion of VCP in routine serum test should significantly improve the diagnostic accuracy of ACS in the early stage. Besides, detection of serum VCP could also be beneficial to patients with ACS due to its informative role in prognosis estimation. In clinical practice, Killip classification is widely used to evaluate the severity of pump failure and guide the therapy selection (45). Nevertheless, the assessment is based on clinical findings, which can be subjective and interfered by respiratory infection symptoms, not to mention that it is unable to reflect the development of VD. On the other hand, existing indicators, such as CK-MB and cTnT for the diagnosis of ACS, or NT-proBNP for the diagnosis of heart failure, are all released passively after ischemia-induced myocardial cell injury, myocardial ventricle wall stress, or tissue hypoxia and therefore could not be applied as specific and valuable prognostic indicators during progression of ACS (46, 47). In this study, two morphological indicators, LVDs and IVS, were also identified to be significantly correlated with the risk of developing VD; however, these are not suitable for clinical application. Blood biochemistry test is currently an important procedure during the clinical evaluation of ACS. As a molecular indicator that can be quantified by the broadly used ELISA, VCP could be easily integrated with some existing biochemical indicators without the requirement of additional pretreatment for the blood samples. More importantly, an advantage of VCP is that it allows a much earlier prediction of prognosis compared to other morphological changes that may only take place in severe phase, which could be helpful in assessing whether some early interventions are needed to prevent poor prognosis of ACS.

The main strengths of our study are 3 folds: (1) we discovered the dual function of serum VCP as it predicts both the development of ACS and its complication. The versatility of VCP suggested its superiority over the current clinical markers; (2) we performed the GWAS and eQTL analyses to explore the potential mechanisms behind the VCP function; (3) we also validated VCP stability under different storage conditions, which might increase the feasibility and practicability of translating VCP into clinical use. However, several limitations should be noted. First, the prognosis prediction role of VCP deserves to be verified in a larger size of samples, since there are currently only 14 serum samples from the “ACS + VD” subgroup, which makes our conclusion lack of confidence. Second, stratification of the VD in patients with ACS based on the degrees of their cardiac dysfunction or physical activity condition was not performed in this study due to the relative small sample size. Hence, further research with more samples consisting of patients with different severities of complications are required to explicit the dynamic expression changes of VCP during the development of VD.

## Conclusion

Overall, the serum level of VCP was found to be positively correlated with the risk of ACS. Higher serum VCP level was significantly related to reduced odds of developing VD in patients with ACS. Serum VCP was stable under different preservation conditions. Our study suggests that VCP is a promising and stable biomarker that can be used to predict both the development of ACS and its complication.

## Data Availability Statement

Publicly available datasets were analyzed in this study. This data can be found here: http://www.ensembl.org/. The datasets generated and analyzed for this study can be obtained by contacting the corresponding authors.

## Ethics Statement

The studies involving human participants were reviewed and approved by Ethics Committee at Zhongshan Hospital, Fudan University. The patients/participants provided their written informed consent to participate in this study.

## Author Contributions

CX and BY performed the experiments, data analysis, and drafted the manuscript. XZ and PG prepared the serum samples. XZ collected the clinical data for all participants. XL, XT, and ZL helped in serum preparation, provided technical assistance in GWAS analysis, and statistical analysis. JG supervised this study. SW and LL conceived and supervised this study and revised the manuscript. All authors contributed to the article and approved the submitted version.

## Funding

This study was financially supported by the National Natural Science Foundation of China (grant nos. 81871527 and 82070285), the Shanghai Health Committee Research Foundation (20194Y0066), the Zhengyi Scholar Foundation of School of Basic Medical Sciences, Fudan University (no. S25-15), and the Fudan Junzheng Scholar Foundation (no. 2193101011003).

## Conflict of Interest

LL, CX, BY, ZL, and XT are inventors on a patent application 202110561991.0 submitted by Fudan University that covers A novel application of serum LMAN2, CAPN-1, and VCP in diagnosing early myocardial ischemia-induced sudden cardiac death. The remaining authors declare that the research was conducted in the absence of any commercial or financial relationships that could be construed as a potential conflict of interest.

## Publisher's Note

All claims expressed in this article are solely those of the authors and do not necessarily represent those of their affiliated organizations, or those of the publisher, the editors and the reviewers. Any product that may be evaluated in this article, or claim that may be made by its manufacturer, is not guaranteed or endorsed by the publisher.
